# Evaluating Novel Chest Compression Technique in Infant CPR: Enhancing Efficacy and Reducing Rescuer Fatigue in Single-Rescuer Scenarios

**DOI:** 10.3390/children12030346

**Published:** 2025-03-10

**Authors:** Marek Solecki, Monika Tomaszewska, Michal Pruc, Magdalena Myga-Nowak, Wojciech Wieczorek, Burak Katipoglu, Basar Cander, Lukasz Szarpak

**Affiliations:** 1 Department of Clinical Research and Development, LUXMED Group, 02-678 Warsaw, Poland; 2 Collegium Medicum, Jan Dlugosz University in Czestochowa, 42-200 Czestochowa, Poland; 3 Department of Emergency Medicine, Medical University of Warsaw, 02-005 Warsaw, Poland; 4 Clinic of Emergency Medicine, Ankara Etlik City Hospital, 06170 Ankara, Turkey; 5 Department of Emergency Medicine, Bezmialem Vakif University, Fatih, 34093 Istanbul, Turkey; 6 Institute of Medical Science, Collegium Medicum, The John Paul II Catholic University of Lublin, 20-950 Lublin, Poland; 7 Henry JN Taub Department of Emergency Medicine, Baylor College of Medicine, Houston, 77030 TX, USA

**Keywords:** infant CPR, chest compression techniques, two-finger technique, two-thumb encircling hands technique, novel compression technique, CPR quality, rescuer fatigue, compression depth, neonatal resuscitation

## Abstract

**Background/Objectives**: Effective infant cardiopulmonary resuscitation (CPR) relies on high-quality chest compressions, yet the optimal technique for single-rescuer scenarios remains debated. Although widely used, the two-finger technique (TFT) is associated with an inadequate compression depth and increased rescuer fatigue. While the two-thumb encircling hands technique (TTHT) provides a superior compression depth, its application in single-rescuer scenarios is impractical. This study evaluates a novel technique (nT) as a potential alternative, aiming to optimize both compression efficacy and rescuer endurance. **Methods**: This randomized crossover study assessed the efficacy of the TFT, TTHT, and nT in a simulated infant CPR setting. Medical students trained in newborn and infant resuscitation performed all three techniques in a controlled environment using a high-fidelity neonatal simulator. We objectively measured and compared key CPR performance metrics, rescuer fatigue, and hand pain among the techniques. **Results**: The nT and TTHT outperformed the TFT in compression depth, rescuer endurance, and overall CPR quality. The nT achieved the highest adequate compression rate (92.4% vs. 78.6% for TTHT and 65.2% for TFT) while minimizing fatigue (RPE: 3.1 vs. 4.5 for TTHT and 6.2 for TFT) and hand pain (NRS: 1.8 vs. 3.9 for TTHT and 5.4 for TFT). TTHT produced the deepest compressions (mean: 44.2 mm vs. 42.9 mm for nT and 38.6 mm for TFT, *p* < 0.001). Rescuer anthropometric factors (sex, weight, and height) affected all techniques similarly, suggesting no inherent advantage based on body characteristics. **Conclusions**: Both the nT and TTHT outperformed the TFT, with the nT demonstrating superior rescuer endurance while maintaining high-quality compressions. Given its ergonomic benefits and effectiveness, the nT emerges as a promising alternative for single-rescuer infant CPR and warrants consideration for future resuscitation guidelines.

## 1. Introduction

While cardiac arrest in infants is not common, it poses the greatest emergency, where cardiopulmonary resuscitation (CPR) needs to be performed promptly and efficiently to improve the chances of survival and neurological condition of the affected child [1,2]. In infants, CPR chest compressions are especially essential because they provide perfusion of the crucial organs during the pause of blood flow. The European Resuscitation Council (ERC) and the American Heart Association (AHA) provide guidelines for infant and child resuscitation based on the best available research [3,4]. These guidelines include instructions about chest compression depth, rate, and technique, among other parameters. However, how chest compressions should be given in this age group is still being studied.

Infants who experience out-of-hospital cardiac arrests (OHCAs) number from approximately 10 to 15 per 100 thousand infants in a year [5,6]. The prognosis in these cases is still very poor, with a 6–10% survival rate to hospital discharge reported and an even smaller number of infants with good neurological conditions. Conversely, in-hospital cardiac arrest (IHCA) incidents are more prevalent in infants, with survival rates ranging from 20% to 40%, contingent upon the etiology, the efficacy of CPR, and the subsequent pediatric intensive care provided [7,8]. With such attention to detail and care needed for infants, this reinforces why CPR techniques need so much focus and why improving methods is such an important area of study.

At present, the ERC and AHA recommend the following two primary methods for performing chest compressions in children and infants: the two-finger technique (TFT) and the two-thumb encircling hands technique (TTHT). The TTHT is best suited for twin rescuers, where both thumbs are used to compress the child’s chest while hands are placed around the child’s torso. On the other hand, the TFT, which is recommended for single rescuers, uses fingers to compress the sternum. Research shows that the TTHT is superior in both defibrillation depth and chest compression, leading to enhanced blood circulation and reduced tiredness in the rescuer. Its main limitation, however, rests in its applicability to single-rescuer situations, especially when used in the context of transitioning between ventilation and chest compressions [9,10].

Previous TTHT vs. TFT studies have suggested that the TTHT has an edge in terms of achieving the optimal compression depth, as well as reducing rescuer fatigue. Jiang et al., for example, stated that the TTHT group yielded a mean incision depth of 42.37 mm while the TFT group had a mean depth of 39.25 mm, along with higher rescuer endurance during extended periods of resuscitation [11]. Even with such results, TFTs are quite common, especially where a single rescuer is required to perform continuous chest compressions and ventilations [12,13,14]. Even so, there is no considerable agreement on the best technique for compressions with infants, hence, further efforts are essential to developing appropriate methods of achieving resuscitation.

This study aims to enhance the existing knowledge by systematically evaluating the efficacy of the TFT, TTHT, and the technique used by the author for chest compression in a controlled simulated infant CPR environment. This study aims to optimize the ideal compression depth, rate, rescuer fatigue, and overall quality of CPR in one-person rescue scenarios.

## 2. Materials and Methods

### 2.1. Study Design and Participants

This study was designed as a randomized crossover trial aimed at comparing the following three infant chest compression techniques: the two-finger technique, the two-thumb encircling technique, and the new author’s technique. The trial was conducted in a controlled simulation environment and adhered to international ethical guidelines for research involving human participants. The study was approved by the Institutional Review Board of the Polish Society of Disaster Medicine (IRB No. 43-2024-0312-IRB).

A crossover design was chosen to minimize inter-individual variability by ensuring that each participant served as their own control, thereby allowing for direct comparisons between the different compression techniques. The study followed the CONSORT guidelines for reporting crossover trials to ensure transparency and reproducibility.

### 2.2. Participants

This study included medical students enrolled in a newborn and infant resuscitation training program, who voluntarily participated in the research. Participants were selected from a cohort of medical students who had successfully completed an accredited neonatal and infant resuscitation course and voluntarily agreed to participate in the study. The inclusion criteria required that participants had undergone prior training in Basic Life Support (BLS) and Pediatric Advanced Life Support (PALS), had no musculoskeletal conditions that could impair their CPR performance, and had not gained advanced clinical experience in neonatal resuscitation (e.g., clinical rotations in neonatology or pediatric intensive care). Those who met the eligibility criteria and provided informed consent were included in the study without additional selection criteria.

Participants were excluded if they had prior advanced clinical experience in neonatal resuscitation, such as rotations in neonatology or pediatric intensive care, or if they reported physical limitations, pain, or injuries that could interfere with performing chest compressions. Participants who did not complete the full training session and study protocol were also excluded from the final analysis.

### 2.3. Setting and Interventions

All participants were recruited from a structured newborn and infant resuscitation training program, a mandatory component of their medical curriculum. Prior to participation, they attended a standardized 60-min theoretical session covering the pathophysiology of neonatal and infant cardiac arrest, CPR guidelines, and the technical aspects of the three studied compression techniques, the TFT, TTHT, and nT.

**Figure 1:**
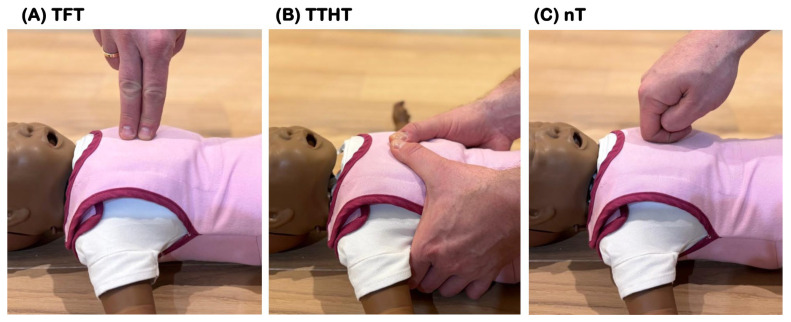
Chest compression techniques used in current study: (**A**) two-finger technique (TFT); (**B**) two-thumb encircling hands technique (TTHT); and (**C**) the novel technique (nT).

The three compression techniques evaluated in the study were as follows:

- The Two-Finger Technique (TFT)—This technique involves placing the tips of two fingers (typically the index and middle fingers) on the lower third of the infant’s sternum to deliver compressions (Figure 1A);
- The Two-Thumb Encircling Hands Technique (TTHT)—In this approach, the rescuer’s both hands encircle the infant’s chest, with the thumbs placed side by side on the lower third of the sternum to provide compressions (Figure 1B);
- The novel technique (nT)—This utilizes the dominant hand for performing chest compressions. In this method, the rescuer’s index finger is actively flexed at the proximal interphalangeal joint, positioning the middle phalanx parallel to the infant’s chest. The external (dorsal) surface of the middle phalanx makes direct contact with the sternum, while the thumb supports the index finger from the opposite side, ensuring stability and controlled force distribution. This unique positioning allows for effective compression delivery with a stable, firm grip while minimizing excessive pressure on the rescuer’s finger joints. The hand remains perpendicular to the infant’s chest throughout the compression cycle, ensuring that force is applied in a direct vertical direction. This approach is designed to optimize compression depth, reduce rescuer fatigue, and enhance overall force control compared to conventional two-finger techniques. The detailed positioning of the fingers and their interaction with the chest wall is illustrated in Figure 1C.

Following the theoretical component, the participants underwent a practical hands-on training session where an experienced instructor demonstrated the techniques and supervised their application on an advanced neonatal manikin (Little Baby QCPR, Laerdal Medical, Stavanger, Norway). To ensure consistency in performance, the students were allowed a practice 30-min session before the experimental phase to familiarize themselves with each technique.

During the experimental phase, a different neonatal simulator (Laerdal Medical SimBaby, Laerdal Medical, Stavanger, Norway) was used to ensure standardization and to minimize the risk of bias associated with evaluating CPR performance on the same simulator used for training. This decision was made to prevent potential learning effects or unconscious adaptation to the previously used manikin, which could influence compression technique, depth, or consistency. By introducing a separate, high-fidelity neonatal simulator specifically for data collection, we aimed to create a controlled and unbiased evaluation environment where performance metrics accurately reflected the effectiveness of each CPR technique, rather than familiarity with a particular device. The experimental simulator was calibrated before each session to maintain measurement accuracy and reliability across all participants. To ensure standardization, the simulator was positioned on a flat, hard surface, and its height was adjusted to two-thirds of the thigh of the rescuer. This setup aimed to optimize ergonomics and provide a consistent frame of reference for chest compression quality assessment.

**Figure 2:**
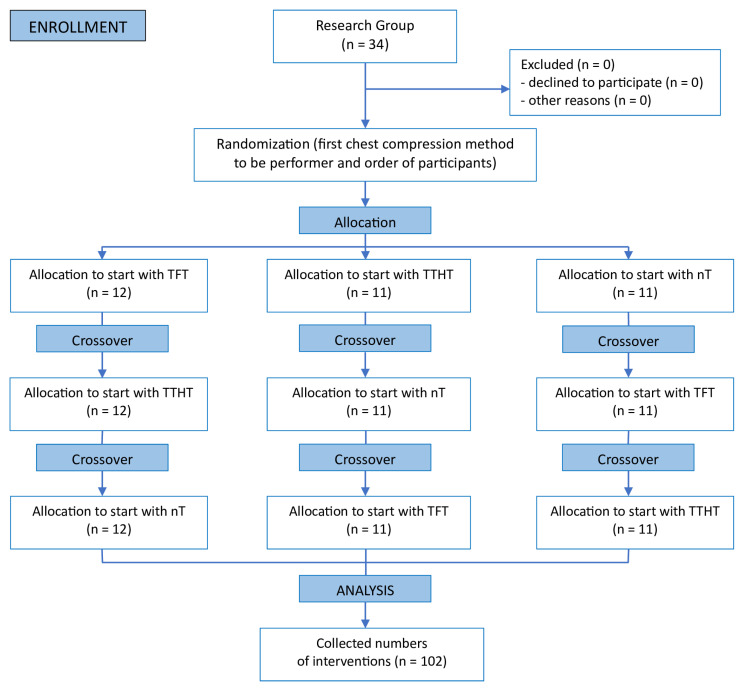
Randomization procedure (TFT: two-finger technique; TTHT: two-thumb encircling hands technique; and nT: the novel technique).

A computer-generated randomization sequence was used to allocate the participants into three groups, with each starting chest compressions with a different technique to minimize order effects and potential learning bias. The randomization was performed using Research Randomizer (www.randomizer.org; accessed on 12 January 2025), a validated tool for generating random allocation sequences. The allocation was concealed from the researchers conducting the assessments until the study commenced to prevent any potential bias. Additionally, the randomization process was carried out by an independent investigator who was not involved in data collection or analysis. The participants were randomly assigned to three groups, with each starting the chest compression sequence with a different technique to minimize order effects and potential learning bias. The first group began with the two-finger technique (TFT), the second with the two-thumb encircling hands technique (TTHT), and the third with the novel technique (nT; Figure 2).

After completing a 2 min continuous chest compression cycle, the participants were given a 15 min rest period to reduce fatigue and maintain performance consistency. Following this break, each participant performed another 2 min compression cycle using a different technique, with the sequence continuing until all three groups had completed all three techniques in a crossover design. This approach ensured that every participant served as their own control, allowing for a direct intra-individual comparison of compression effectiveness, rescuer fatigue, and technique efficiency while minimizing variability due to participant skill levels.

### 2.4. Outcomes

The primary outcomes of this study focused on evaluating the quality and effectiveness of chest compressions based on key CPR performance metrics. These included the mean compression rate, the adequate compression rate—defined as the percentage of compressions within the recommended range of 100–120 compressions per minute (CPMs)—the mean compression depth, and the proportion of compressions achieving an adequate depth (≥40 mm) in accordance with the ERC and AHA guidelines. Additionally, full chest recoil was assessed as the percentage of compressions allowing for complete chest relaxation, ensuring minimal coronary occlusion.

To assess the consistency and accuracy of hand positioning, the QCPR system integrated into the simulator automatically recorded and analyzed hand placement relative to the center of the sternum, providing real-time feedback on any deviations. Furthermore, an independent researcher supervised the sessions and verified the results to ensure measurement consistency. All evaluations followed standardized criteria, with structured verification procedures in place to minimize the risk of assessment bias.

The secondary outcomes comprised the subjective and physiological reactions of the rescuers towards the varying techniques of compression. The participants were asked to rate the ease of compression in order to assess the perceived effort expended using each approach. The fatigue level was self-reported using the Borg Rating of Perceived Exertion (RPE) Scale, while hand pain was assessed using a Numerical Rating Scale (NRS) from 0 to 10. An overall score measuring performance was computed by objectively assessing quality CPR performance alongside the subjective ratings to determine the efficiency and burden of each technique with respect to the rescuer’s effort. The total score was calculated based on key CPR performance indicators measured by the Laerdal QCPR system. This includes compression depth (optimal range: 5–6 cm), compression rate (100–120/min), full chest recoil, and correct hand positioning. Each parameter contributes to the overall score, reflecting the rescuer’s performance accuracy. The score is expressed as a percentage, where higher values indicate a better adherence to CPR guidelines.

Using the integrated feedback system of the neonatal simulator, outcome measures were collected in real time, ensuring that all participants received accurate and impartial data collection.

### 2.5. Sample Size

The sample size was based on a power analysis from prior randomized crossover studies examining various infant chest compression techniques [11,14,15]. Due to the within-subject design, where each person acts as their own control, so the sample size required for maintaining statistical precision is lower. Prior studies have reported a mean difference of ~3.0 mm (SD = 2.5 mm) between techniques, so compression depth was selected as the primary outcome measure [11]. Using G*Power 3.1, a repeated-measures ANOVA with α = 0.05, power = 80%, and effect size f = 0.25 indicated that 30 participants were required. This aligns with similar studies where 20–40 participants yielded statistically significant results [14]. The crossover design reduces between-subjects variations and improves the quality of the study, satisfying the condition that 34 participants performing all three techniques are more than enough to detect important differences in the compression depth, rate, and fatigue of the rescuer.

### 2.6. Statistical Analysis

The R Studio (Version 2024.12.0 + 467, R Foundation for Statistical Computing, Vienna, Austria) was chosen for statistical operations, which was compiled by the R Foundation for Statistical Computing, Vienna, Austria. For reporting the summary statistics of continuous variables, we provided mean values together with standard deviation (SD). In cases where percentages are reported with mean ± SD (e.g., adequate compression rate), the SD reflects the variability between participants rather than a statistical confidence interval. For some continuous data, the Shapiro–Wilk test was used for benchmark normality, and for variance, Levene’s test was executed. Major perceived outcome measures, like perceived ease of chest compression level, fatigue level, and hand pain, were self-rated on a 10-point Likert scale, and a non-parametric Friedman test was utilized, as these measures did not fluctuate normally. Post hoc pair-wise Wilcoxon signed-rank tests with Bonferroni adjustments were calculated for multiple comparisons after the Friedman test, as post hoc tests assess if a significant difference can be found in the Friedman test. Normality was assumed for continuous variables that passed one-way analysis of variance (ANOVA) tests, and was employed to check differences among the three different techniques. If only one pair of the three pairs (1 and 2, 1 and 3, and 2 and 3) showed statistically significant differences, then independence t-test was used between the two groups. Multi-variate Welch’s correction was used in the presence of heterogeneity of variance. These included, but were not limited to, Total Score, Ease of Compression, Degree of Fatigue, Pain in the Hand, Positioning of the Hand, relaxation level granulation, standing in a condition, Mean Depth, and so on to evaluate the various methods of compression and their effectiveness and usability for each of the methods. To explore the correlation between rescuer’s physical features (gender, body weight, and height) and the effectiveness of compression, the Pearson correlation coefficient was calculated for respective normal data, while the Spearman rank correlation coefficient was used for non-normal data. These analyses examined the influence of family factors on the Total Score, Ease of Compression, Degree of Fatigue, Pain in the Hand, Positioning of the Hand, Full Relaxation of the Chest, Adequate Compression, and Mean Depth of the Compression. Just like other statistical analyses, our tests were two-tailed and had a *p*-value threshold of less than 0.05. Bonferroni adjustment was also used to reduce the effect of multiple comparisons where it was required. These analyses were performed within the R studio software, and the relevant statistical packages were incorporated for the tests, algorithms, and analyses in compliance with the standards of methodology and reproducibility.

## 3. Results

A sample consisting of 34 medical students was recruited for the study, out of which 14 were males (41.2%) and 20 were females (58.8%). Their average age was 21.1 years (SD = 2.6, range: 18–29 years). The average body weight of participants was 71.1 kg (SD = 16.2, range: 44–102 kg), while the average height was 171.3 cm (SD = 10.2, range: 155–190 cm).

The males had a mean weight of 80.6 kg (SD = 11.7), and their mean height was 181.1 cm (SD = 4.4). In comparison, females had a mean weight of 60.2 kg (SD = 13.2), while their average height was 165.3 cm (SD = 6.6). The age, weight, and height of participants were not normally distributed, which can be expected from a sample of people who are undergoing medical training.

All the volunteers were trained in basic life support (BLS) and pediatric advanced life support (PALS) as a part of the medical school curriculum. No participant stated any medical condition which could impair physically one’s ability to perform CPR, thus guaranteeing a relatively equal level of fitness to the cohort sample.

### 3.1. Chest Compression Quality Outcomes

We performed a comparative analysis of three chest compression procedures (TFT, TTHT, and nT) across several performance criteria (Table 1).

**Figure 3:**
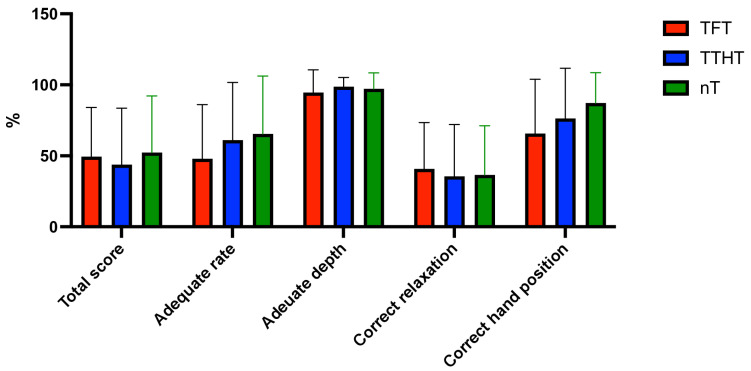
Chest compression quality among research groups.

The total chest compression (CC) score of the procedures showed no notable variations (*p* = 0.654, ANOVA), and pairwise comparisons demonstrated no statistical significance (all *p* > 0.05). The average compression rate differed among approaches (*p* = 0.204, ANOVA), with the TTHT demonstrating the lowest rate. Subsequent analyses indicated significant differences between the TFT and TTHT (*p* = 0.039), as well as between the nT and TTHT (*p* = 0.034; Figure 3). The percentage of sufficient compression rates (100–120/min) was greatest in the nT (65.39 ± 40.75%); however, the overall ANOVA outcome was not significant (*p* = 0.184). A post hoc analysis revealed a significant difference between the TFT and nT (*p* = 0.029). The average compression depth varied significantly between approaches (*p* < 0.001, ANOVA), with the TTHT yielding the greatest depth. Post hoc comparisons revealed significant differences between the TFT and TTHT (*p* < 0.001) and between the TFT and nT (*p* < 0.001), whereas the comparison between the TTHT and nT (*p* = 0.234) was not significant. The percentage of sufficient depth was greatest in the TTHT (98.72 ± 6.43%); however, intergroup differences were not statistically significant (*p* = 0.358, ANOVA). The overall chest recoil percentage exhibited no significant difference across approaches (*p* = 0.808, ANOVA).

The incidence of proper hand positioning was highest in the nT (87.24 ± 21.38%), with statistically significant variations observed among approaches (*p* = 0.031, ANOVA). Post hoc analyses revealed a significant difference between the TFT and nT (*p* = 0.011), although comparisons between the TFT and TTHT (*p* = 0.263) and the TTHT and nT (*p* = 0.145) were not statistically significant.

**Figure 4:**
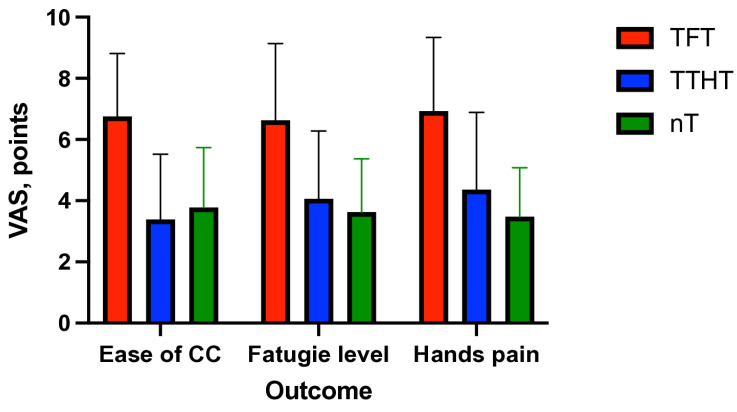
Effect of compression on fatigue and pain complaints.

The TTHT (3.39 ± 2.13) and nT (3.78 ± 1.96) were rated as being significantly easier to perform compared to the TFT (6.75 ± 2.06; Figure 4). Post hoc analysis confirmed significant differences between the TFT vs. the TTHT (*p* < 0.001) and the TFT vs. the nT (*p* < 0.001), while no significant difference was found between the TTHT and nT (*p* = 0.35).

A significant effect of compression technique on fatigue levels was observed (*p* < 0.001, ANOVA;). The TFT induced the highest fatigue (6.63 ± 2.51), followed by the TTHT (4.06 ± 2.22) and nT (3.63 ± 1.74). Post hoc comparisons confirmed that the TFT resulted in a significantly greater fatigue than the TTHT (*p* < 0.001) and nT (*p* < 0.001), whereas the difference between the TTHT and nT (*p* = 0.26) was not statistically significant.

Significant differences in hand pain were also identified (*p* < 0.05, ANOVA), with the TFT being associated with the highest reported discomfort (6.93 ± 2.41), followed by the TTHT (4.36 ± 2.53) and nT (3.48 ± 1.60). Post hoc analyses confirmed that the TFT caused significantly more hand pain than the TTHT (*p* < 0.001) and nT (*p* < 0.001). Additionally, the TTHT resulted in significantly greater pain than nT (*p* = 0.049; Table 2).

### 3.2. Influence of Gender, Weight, and Height on Chest Compression Performance

For the participants who were using the TFT technique, male responders had a weak positive correlation with adequate chest compression (r = 0.30) and full chest relaxation (r = 0.27; Table 3). This indicates that males improved the utilization of compression efficiency and recoil as opposed to their female counterparts. Furthermore, the negative correlation with hand pain (r = −0.18) indicates that the pain levels in the hands were lower among male responders. A positive correlation between body weight and perceived chest compression ease (r = 0.26) shows that people with a larger body had less difficulty while performing the chest compressions. However, weight had no or very little effect on average compression depth (r = −0.003) and average performance score (r = −0.17) which indicates that the ability to perform compressions did not depend greatly on body size. On the other hand, height had a positive correlation with compression ease (r = 0.33) and adequate compression rate (r = 0.31), suggesting that individuals with a greater height were able to perform more efficient compressions. Yet, a negative correlation with correct hand position (r = −0.11) implies that the proper hand positioning may have been harder to achieve for taller responders.

For the TTHT, the participants’ scores were rather precisely positively correlated with the total performance score (r = 0.29), which means that men showed an improvement in their overall effectiveness. A view correlating with the opposite of this explanation is the correlation of fatigue levels, r = −0.16, which indicates that male rescuers were less exhausted than female participants. Body weight positively correlated with total score (r = 0.28) and mean compression rate (r = 0.29), meaning that people with an increased body mass performed more effective compressions.

On the other hand, weight was negatively associated with ease of compression (r = −0.20) and level of fatigue (r = −0.21), suggesting that heavier rescuers found compressions to be more difficult. Height positively influenced the performance, as well as the total score (r = 0.38) and the mean compression rate (r = 0.21), so it can be said that more efficient people are taller, although a negative correlation with mean compression depth (r = −0.30) indicates that subjects of a taller stature tended to have problems with endurance of compression depth. There is a lesson to learn from this; increasing either body mass or height will produce an enhanced compression performance, but also create ergonomic problems that will result in inconsistences, as well as wear and tear of the rescuer.

While using the nT method, gender had a weak positive correlation with the ease of compression, which was r = 0.16, and full chest relaxation, which was r = 0.16. This implies that male participants reported that compressions were easier to perform and showed greater recoil. Nonetheless, a negative correlation with proper hand positioning, which was r = −0.10, implies that males might have had issues with performing proper hand placement. Body weight showed a negative correlation with levels of fatigue, which were r = −0.25 and r = −0.37, which means that heavier people were less fatigued and less pained. On the flip side, a positive correlation with full relaxation, which was r = 0.21, implies that increased body mass was a factor in improved recoil performance. Nevertheless, a negative correlation with adequate compression rate, which was r = −0.22, suggests that participants who had elevated body weight struggled with meeting the proper compression frequency. A positive correlation of r = 0.21 with the ease of compression was found with taller persons, which means that height can be beneficial for compression technique. But these were accompanied by negative correlations with the adequate rate, which was r = −0.21, and the proper hand positioning, which was r = −0.11, which means that taller rescuers had more difficulties with maintaining the proper compression rate and hand placement.

## 4. Discussion

The present study systematically evaluated the efficacy of three infant chest compression techniques—the two-finger technique (TFT), the two-thumb encircling hands technique (TTHT), and the author’s novel technique (nT)—in a simulated infant CPR setting. Even though the TFT continues to be the preferred technique for single rescuers, as it allows for an effortless transition from compressions to ventilation, it is known widely that the TFT is insufficient in both achieving an adequate compression depth and minimizing rescuer fatigue. The TTHT is generally recommended for two-rescuer CPR because it enables a greater depth and hemodynamic efficiency during chest compressions [15,16,17]. However, it is not feasible for a lone rescuer. Previous attempts have been made to show that both the TTHT and nT are superior to the TFT in terms of accomplishing an adequate compression depth alongside minimizing rescuer fatigue and pain, as well as achieving high-quality CPR metrics. These findings point towards the nT’s advantage as being more ergonomic, as well as effective for single-rescuer CPR, closing the gap between the adaptable nature of the TFT and the depth advantages of the TTHT.

According to the present ERC and AHA guidelines, the compression depth must cover a minimum of one-third of the infant’s anterior–posterior chest diameter, which alters to around 40 mm [18]. The TTHT has persistently shown better compression results, but restrictive single-performer scenarios remain a hurdle [19,20,21]. In our study, the TTHT presented the deepest compressions, and we found it to be in line with previous reports noting that the TTHT has acquired a favorable thumb strength for operating deeper with a throttle stability above head height [11,22].

Compression utilizing the thumb and the middle phalanx of the index finger, which is referred to as the novel technique (nT), attained comparably deep compression with the TTHT (*p* = 0.234) while minimizing hand fatigue and pain beyond statistical significance (*p* < 0.001 vs. TFT). These results correspond to the complaints of depth recommendation failure advanced by TFU. In addition, the nT permits a much better force distribution and could lower concerns with regard to excessive metacarpophalangeal loading, which is the result of the TFT [23]. These findings add support to the argument which poses that the nT can be presented as an alternative in single-performer infant CPR instead of squeezing patients with a set depth while increasing the imposition of engagement.

A major contributor to rescuer fatigue while performing CPR for long periods is persistence in performing compressions. Earlier research states that the TFT results in greater fatigue (RPE: 6.63 vs. 3.63–4.06) and hand discomfort (NRS: 4.7 vs. 2.2–2.5) than the thumb techniques [24]. Our data corroborate this evidence by showing that rescuer fatigue with the nT is less than that with the TFT, while compression quality is higher than that provided by the nT.

Aligning force through the rescuer’s dominant hand while supporting compression with the thumb does inadvertently bring some biomechanical benefits of the nT. In contrast with Barcala-Furelos et al. [25], Smereka et al. showed that vertical two-thumb techniques resulted in more body weight being shifted towards the head and a greater stability resulting from exertion being minimized. Such benefits for single rescuers are of critical focus, as the quality of CPR compressions performed over prolonged periods is the difference between life and death [26,27]. In a situation where single-rescuer-operated nT CPR techniques are the primary focus, these reduced discomforts during the nT may increase rescuer compliance and effort in emerging countries.

Our analysis indicates that gender, body weight, and height influenced all three techniques similarly, with no method demonstrating a reduced anthropometric impact. Consistent with Haque et al., males exhibited a slightly greater efficiency with the TFT, likely due to a greater upper-body strength, whereas taller individuals encountered positioning challenges across all techniques [28,29,30]. These findings underscore the need for adaptable CPR techniques that optimize performance across diverse rescuer profiles. The nT’s improved force distribution and stability may offer a universal advantage, though additional studies are needed to validate its effectiveness across broader rescuer demographics.

Despite the TTHT’s superior hemodynamic outcomes, its practicality in single-rescuer CPR remains limited due to difficulties in transitioning between compressions and ventilations. Our findings, along with recent work by Smereka et al. and Ladny et al., suggest that the nT bridges this gap, offering a TTHT-like depth with a TFT-like adaptability [29,31]. Given its ergonomic advantages and reduced rescuer fatigue, integrating the nT into CPR training programs could improve compliance with depth guidelines (≥40 mm) and enhance rescuer endurance.

Furthermore, skill retention remains a major challenge in pediatric CPR [23]. Training programs should explore the nT’s potential in reducing skill decay, given its intuitive design and improved rescuer comfort. The reduction in fatigue and hand pain associated with the nT could encourage greater adherence to CPR guidelines, particularly in high-stress, prolonged resuscitation scenarios.

### Limitations

This research has several limitations. While manikin-based research is broadly endorsed in CPR studies, it fails to accurately emulate the biomechanical characteristics of human tissue or the psychological and environmental variables present in actual resuscitation scenarios. The study participants were medical students with consistent training backgrounds, perhaps restricting the generalizability of the findings to lay rescuers or seasoned professionals. The controlled simulation environment fails to consider the variety of clinical settings, including patient-specific anatomical variations and environmental stressors that may affect CPR performance. Subsequent investigations ought to concentrate on substantiating the efficacy of the novel two-thumb technique (nT) in practical environments, encompassing both in-hospital and out-of-hospital cardiac arrest situations, where elements such as rescuer fatigue, fluctuating response times, and situational limitations may impact technique effectiveness. Additionally, subsequent research should evaluate the adaptability of the nT among diverse rescuer populations, encompassing healthcare professionals with differing experience levels, paramedics, and non-medical responders, as well as its relevance to specific patient demographics, including preterm neonates and older infants. Examining long-term skill retention is essential, as previous studies indicate that CPR performance deteriorates over time without consistent reinforcement. Furthermore, comparison studies ought to investigate the performance of the nT in relation to other new methodologies, such as the vertical two-thumb approach, which has demonstrated potential in enhancing compression quality while alleviating rescuer fatigue. Subsequent research should investigate the practicality of integrating the nT into standardized training regimens and its effect on adherence to resuscitation recommendations across various clinical and educational environments.

## 5. Conclusions

This study’s findings indicate that the nT and TTHT surpass the TFT for compression depth, rescuer endurance, and overall compression quality. Of the three approaches, the nT demonstrated the least rescuer fatigue and hand discomfort while attaining the highest rate of acceptable compressions, but the TTHT yielded the greatest compression depth, occasionally above the required range. The investigation indicated that anthropometric parameters, including sex, weight, and height, exerted a consistent influence across all procedures, with no method providing a specific advantage depending on body attributes. This indicates that, although individual rescuer characteristics may impact CPR effectiveness, individuals do not necessarily prefer one approach over another; instead, they influence all procedures similarly.

## Figures and Tables

**Table 1:** Chest compression quality outcomes.

Parameter	Chest Compression Technique, Mean (SD)			ANOVA Statistical Test Results			
	TFT	TTHT	nT	All Techniques	1 vs. 2	1 vs. 3	2 vs. 3
Total score, %	49.42 (34.59)	43.75 (39.87)	52.30 (39.86)	0.654	0.539	0.755	0.387
Mean rate, CC/min	113.64 (16.82)	108.24 (12.69)	113.58 (12.05)	0.204	0.039	0.982	0.034
Adequate rate (100–120), %	47.91 (38.12)	61.03 (40.67)	65.39 (40.75)	0.184	0.084	0.029	0.547
Mean depth, mm	48.36 (6.36)	55.60 (9.27)	53.69 (5.28)	<0.001	<0.001	<0.001	0.234
Adequate depth, %	94.54 (16.04)	98.72 (6.43)	97.15 (11.26)	0.358	0.183	0.268	0.381
Full relaxation, %	40.81 (32.68)	35.57 (36.50)	36.57 (34.60)	0.808	0.343	0.513	0.801
Correct hands position, %	65.72 (38.21)	76.30 (35.38)	87.24 (21.38)	0.031	0.263	0.011	0.145

Legend: TFT: two-finger technique; TTHT: two-thumb encircling hands technique; and nT: the novel technique.

**Table 2:** Impact of chest compression technique on rescuer comfort.

Parameter	Chest Compression Technique, Mean (SD)			Statistical Test Results			
	TFT	TTHT	nT	All Techniques	1 vs. 2	1 vs. 3	2 vs. 3
Ease of chest compression	6.75 (2.06)	3.39 (2.13)	3.78 (1.96)	<0.05	<0.001	<0.001	0.35
Fatigue level	6.63 (2.51)	4.06 (2.22)	3.63 (1.74)	<0.001	<0.001	<0.001	0.26
Hand pain	6.93 (2.41)	4.36 (2.53)	3.48 (1.60)	<0.05	<0.001	<0.001	0.049

Legend: TFT: two-finger technique; TTHT: two-thumb encircling hands technique; and nT: the novel technique.

**Table 3:** Correlation between anthropometric parameters and chest compression quality.

Parameter	Gender Differences	Effect of Body Weight	Impact of Height
**TFT**			
Total compression score	0.063	−0.167	−0.044
Mean rate	0.107	0.194	−0.074
Adequate rate	0.302	0.260	0.306
Mean depth	0.136	−0.003	0.153
Adequate rate	0.009	0.043	−0.063
Full relaxation	0.265	0.176	0.246
Correct hands position	−0.073	−0.038	−0.112
Ease of chest compression	0.283	0.260	0.330
Fatigue level	−0.146	−0.159	−0.113
Hand pain	−0.184	−0.070	0.028
**TTHT**			
Total compression score	0.286	0.282	0.378
Mean rate	0.164	0.289	0.214
Adequate rate	−0.063	−0.046	0.052
Mean depth	−0.077	−0.148	−0.301
Adequate rate	0.153	0.098	−0.030
Full relaxation	0.204	0.200	0.231
Correct hands position	0.075	0.107	0.113
Ease of chest compression	−0.044	−0.195	−0.135
Fatigue level	−0.163	−0.208	−0.167
Hand pain	0.096	−0.016	0.102
**nT**			
Total compression score	0.029	0.118	−0.028
Mean rate	−0.119	0.004	−0.175
Adequate rate	0.003	−0.224	−0.207
Mean depth	0.062	0.034	0.111
Adequate rate	−0.105	0.011	−0.086
Full relaxation	0.158	0.213	0.210
Correct hands position	−0.103	0.072	−0.111
Ease of chest compression	0.157	−0.123	0.213
Fatigue level	0.074	−0.249	−0.123
Hand pain	0.047	−0.366	−0.009

## Data Availability

The data that support the findings of this study are available on request from the corresponding author (L.S.).

## Abbreviations

The following abbreviations are used in this manuscript:

AHA	American Heart Association
BLC	Basic Life Support
CC	Chest Compression
CPR	Cardiopulmonary Resuscitation
ERC	European Resuscitation Council
IHCA	In-Hospital Cardiac Arrest
NRS	Numerical Rating Scale
OHCA	Out-of-Hospital Cardiac Arrest
PALS	Pediatric Advanced Life Support
RPE	Rating of Perceived Exertion
TFT	Two-Finger Technique
TTHT	Two-Thumb Encircling Hands Technique
nT	Novel Compression Technique
